# Skeletal age assessed by TW2 using 20-bone, carpal and RUS score systems: Intra-observer and inter-observer agreement among male pubertal soccer players

**DOI:** 10.1371/journal.pone.0271386

**Published:** 2022-08-23

**Authors:** Paulo Sousa-e-Silva, Manuel J. Coelho-e-Silva, Andre Seabra, Daniela C. Costa, Diogo V. Martinho, João P. Duarte, Tomás Oliveira, João Gonçalves-Santos, Inês Rodrigues, Luis P. Ribeiro, António J. Figueiredo, Jan M. Konarski, Sean P. Cumming, Robert M. Malina

**Affiliations:** 1 University of Coimbra, FCDEF, Coimbra, Portugal; 2 University of Coimbra, CIDAF, Coimbra, Portugal; 3 Portugal Football School, Portuguese Football Federation, Lisbon Portugal; 4 Faculty of Sport, University of Porto, CIAFEL, Porto, Portugal; 5 Poznań University of Physical Education, Theory of Sports Department (Sport Science), Poznań, Poland; 6 Department of Health, University of Bath, Bath, United Kingdom; 7 Department of Kinesiology and Health Education, University of Texas, Austin, Texas, United States of America; 8 University of Louisville, School of Public Health and Information Sciences, Louisville, Kentucky, United States of America; Nottingham Trent University, UNITED KINGDOM

## Abstract

The purpose of this study was to determine intra- and inter-observer agreement for the three skeletal ages derived from the TW2 method among male pubertal soccer players. The sample included 142 participants aged 11.0–15.3 years. Films of the left hand-wrist were evaluated twice by each of two observers. Twenty bones were rated and three scoring systems used to determine SA adopting the TW2 version: 20-bone, CARPAL and RUS. Overall agreement rates were 95.1% and 93.8% for, respectively, Observer A and Observer B. Although, agreement rates between observers differed for 13 bones (5 carpals, metacarpal-I, metacarpal-III, metacarpal-V, proximal phalanges-I, III and V, distal phalanx-III), intra-class correlationa were as follows: 0.990 (20-bone), 0.969 (CARPAL), and 0.988 (RUS). For the three SA protocols, BIAS was negligible: 0.02 years (20-bone), 0.04 years (CARPAL), and 0.03 years (RUS). Observer-associated error was not significant for 20-bone SA (TEM = 0.25 years, %CV = 1.86) neither RUS SA (TEM = 0.31 years, %CV = 2.22). Although the mean difference for CARPAL SAs between observers (observer A: 12.48±1.18 years; observer B: 12.29±1.24 years; t = 4.662, p<0.01), the inter-observer disagreement had little impact (TEM: 0.34 years: %CV: 2.78). The concordance between bone-specific developmental stages seemed was somewhat more problematic for the carpals than for the long bones. Finally, when error due to the observer is not greater than one stage and the replicated assignments had equal probability for being lower or higher compared to initial assignments, the effect on SAs was trivial or small.

## Introduction

Growth, maturation and development are central processes in long-term participation of children and youth in competitive sports. The preceding processes are not synonymous or interchangeable. Growth involves quantitative changes in body size, proportions, shape and composition [1]. Development implies changes in behavioral domains: cognitive, emotional, social and motor. Finally, maturation marks progress towards the adult state which varies with the biological system: dental, sexual, somatic and skeletal. In the context of youth sports, the concepts are implicit to the long-term athletic development model [2]. Two indicators of biological maturation are commonly used in studies of adolescent athletes. In boys, sexual maturation includes genital and pubic hair development that are limited to pubertal years. Skeletal age (SA) requires standard radiographs of the hand and wrist and is generally considered the preferred indicator as it can be assessed through the first two decades of life [3]. Several protocols are available to determine SA [4–8]. They are similar in principle and require a radiograph of the hand-wrist. Advancements in technology have reduced exposure to radiation to about 0.001 millisievert (mSv), which is equivalent to three hours of television [3]. Briefly, the Greulich-Pyle [4] and Fels [5] methods were developed on American children and adolescents, while the Tanner-Whitehouse [6] is originally based on British youth and was subsequently modified [7,8].

Observations on Portuguese soccer players aged 11–12 years suggest that youth delayed and advanced in terms of skeletal maturity status based on Fels SAs were equally represented, whereas among players 13–14 years late maturing players were underrepresented whereas those classified as average and early were over-represented [9]. The previous confirms soccer as highly selective and the literature suggests a gradient in body size among Portuguese male soccer players aged 13.0–14.1 years of age [10]: those selected for a regional team were taller, heavier, advanced in AS given by the Fels method and had more playing experience than teammates who were not selected. Meantime, among French youth players [11], those who signed a professional contract and played at least one game as a professional were significantly taller and heavier and had a higher estimated aerobic power at baseline (~13 years) compared to peers who did not sign a professional contract, although the groups did not differ in skeletal maturity assessed by the Greulich Pyle protocol. Finally, a survey of the skeletal maturity status of Serbian soccer players aged 14 years using the Tanner-Whitehouse method, more precisely the radius-ulna-short bones (RUS), noted that late maturing players were more likely to attain a professional career compared to early maturing peers [12].

The above cited youth soccer literature produced different results which highlight the need to discuss the generalization of studies based on concurrent methods of SA assessment. In fact, results may reflect variation in the methods to determine SA and/or specific characteristics of youth soccer in Portugal, France and Serbia. The literature already examined the agreement of concurrent protocols for SA determination, particularly during pubertal years overlapping to selection, specialization into playing positions [10] and vulnerability to sport injuries [13]. For example, the SAs of 40 male Spanish soccer players aged 12.5–16.1 years were assessed with the TW3 and Fels methods [14]. A consistent trend for lower SAs with the TW3 RUS compared to Fels was evident. More recently [15], two versions of TW RUS method (TW2 versus TW3) were compared in a large international sample of male soccer players aged 10.9–17.9 years. Across the CA range of the sample, TW3 RUS SAs were consistently lower than TW2 RUS SAs. The preceding studies have implications for the classification of youth players by maturity status. Advances in digital imaging technologies combined to research dealing with machine learning have led to the emergence of informatic applications that automatically estimate SA from digitalized radiographs [16]. Meantime, sonography has been proposed as an alternative non-invasive method for determining SA [17]. The preceding includes the operator, while obtaining the image, as an additional source of error.

Taking into account the preceding, error is a central issue in determination of SA. In the study of Spanish players [14], intra-observer differences for Fels and TW3 SAs fluctuated 0.1 to 0.4 years, while technical errors of measurements were small: Fels SA was 0.04 year, TW3 SA was 0.06 year. The objective of the present study was to evaluate intra-observer and inter-observer agreements for the SAs derived by TW2 method among male adolescent soccer players: 20-bone protocol (TW2 20 bone SA), carpals (Carpal SA), and 13 long bones (RUS SA). It is hypothesized that even trained observers produce errors in the assessment of TW2 SAs.

## Materials and methods

### Procedures

The present study is derived from the *PRONTALSPORT Project (Growth*, *maturation and athletic performance in pubertal athletes)*. The project followed the ethical standards established for sports sciences [18] and was approved by the *Ethics Committee for Sports Sciences* by the *University of Coimbra* (CE/FCDEF-UC/00122014). Participants were recruited from clubs of Portuguese Midlands having a written agreement with *University of Coimbra*. Parents of the players signed an informed consent, while the players provided assent. They were informed that their participation was voluntary and they could withdraw at any time. All data were collected within a 2-week period in the *Coimbra University Stadium* for anthropometry and posterior-anterior radiographs of the left hand-wrist were obtained on the same day at a certified clinic.

### Sample

The sample included 142 male adolescent soccer players aged 11.0–15.3 years. All participants were registered in the *Portuguese Football Federation* as infantiles and initiates. The clubs competed in a 9-month tournament (from middle September until late May). In general, clubs trained 3–5 sessions per week (90–120 minutes) and competed once per week (usually on Saturdays or Sundays).

### Chronological and skeletal ages

CA was calculated as the difference between birthdate and the date of the visit to the clinic. The films of the left hand-wrist were evaluated twice by each of the two observers. Observer A (first author) completed a 3-year Bsc in Sport Sciences in addition to a 2-year Msc in Youth Sports including a 27-hour course dealing with biological maturation. Subsequently, enrolled in the PhD programme and already complete a 45-hour training in the assessment of skeletal age that includes 100 assessments using concurrent methods to determine SA. Before assessing the x-rays of the current study, over the past four years determined SA of more than 1000 cases. The second author is Professor at the University of Coimbra over the past 27 years and was trained by the last author in the determination of SA more than 20 years ago and already assessed more than 5000 films using Greulich Pyle, Tanner-Whitehouse and Fels protocols. Repeated assessments by each of the two observers were obtained after one month.

The TW method—version 2 (TW2) was used to assess skeletal age [7]. The method is based on matching a specific bone on the radiograph with the verbally described criteria for specific stages for the bones. Twenty bones were rated: 13 long bones (radius, ulna, the metacarpals and the proximal, middle and distal phalanges of the first, third and fifth digits) and seven carpals (excluding the pisiform. Stages were essentially the same of the original TW version [6].

The three scoring systems are specific to each SA in the TW2 version: 20-bone, CARPAL and RUS. A specific point score is assigned to each stage for each individual bone. The scores for each bone are summed to give a skeletal maturity score, which ranges from zero (immaturity) to 1000 (maturity). The CARPAL and the RUS bones were somewhat arbitrarily weighted so that each contributed 50% to the total skeletal maturity score and the overall differences between bones within each group were minimized. Finally, sex-specific tables convert the total score at a particular system (20-bone, RUS and CARPAL) into an individual SA. As noted, 1000 points indicates the skeletally mature state and an SA is not assigned for individuals who are skeletally mature [3].

### Analyses

Frequencies for bone-specific developmental stages were presented separately for each occasion (time-moment 1; time-moment 2) for observer A and observer B. Rates of intra-observer agreement were calculated for each individual bone and for the total of observations (142 participants multiplied by 20 bones, 2840 observations). Discrepancies for stages between time-moments were noted as -2, -1, +1, +2 as time moment 2 minus time moment 1). Intra-observer mean differences were also calculated using paired t-tests, separately for bone-specific scores (points) and also for the three systems (20-bone, RUS, CARPAL) and for respective SAs. The preceding was done separately for observer A and observer B. Based on time-moment 2 for each observer, similar analyses were done to examine inter-observer variation in assessments. Technical errors of measurement (TEM), coefficients of variation (%CV) and intra-class correlation coefficients (ICC) were calculated. The magnitude effect was calculated using d-values [19] and interpreted as follows [20]: d<0.20 (trivial), 0.20<d<0.60 (small), 0.60<d<1.20 (moderate), 1.20<d<2.00 (large), 2.00<d<4.00 (very large), and >4.00 (nearly perfect). The analyses using SAs (20-bone SA, Carpal SA, RUS SA) as dependent variables were limited to participants who were not skeletally mature. Significance level was set at 5%. Analyses were performed using the Statistical Package for the Social Sciences version 26.0 (SPSS Inc., IBM Company, Armonk, NY, USA) and GraphPad Prism (version 5 for Windows, GraphPad Software, San Diego California USA, www.graphpad.com).

## Results

Developmental stages for each of the 20 bones at time moment 1 and time moment 2 for each observer are summarized in Table 1. Agreement was 95.1% and 93.8% for, respectively, Observer A and Observer B. Intra-observer error assessed as the difference between time-moment 2 minus time-moment 1 was equally distributed: 70 negative (2.5%) and 69 positive (2.4%) for observer A; and 91 negative (3.2%) and 85 positive (3.0%) for observer B. Technical errors of measurements, coefficients of variation and intra-class correlations for Observer A are summarized in Table 2. For the 20-bone system, mean difference between time moments was significant for the capitate (t = 2.022, p<0.05), although the CV was less than 5% and ICC was 0.823. The ICC fluctuated between 0.823 and 0.993 for, respectively, the capitate and distal phalanx-I; the coefficient was 0.997 (TEM = 8.01, %CV = 0.95) for the 20-bone score. In the CARPAL protocol, the capitate was again the single bone presenting an intra-observer mean difference (t = 2.022, p<0.05; TEM = 6.41, %CV = 3.03; ICC = 0.834). In contrast, there was negligible variation in the CARPAL score (TEM = 8.99, %CV = 0.97; ICC = 0.993). For the RUS protocol, mean differences were not significant and the ICC coefficient for the RUS score was 0.997 (TEM = 13.92, %CV = 2.60). Similarly, intra-observer agreement for observer B on the three scoring systems is summarized in Table 3. Overall, ICC scores were acceptable for each system: 20-bone (TEM = 9.68, %CV = 1.15; ICC = 0.996), CARPAL (TEM = 12.95, %CV = 1.32; ICC = 0.990), RUS (TEM = 19.96, %CV = 3.68; ICC = 0.994). Significant intra-individual mean differences were noted for medial phalanx-V (t = -3.754, p<0.01; TEM = 0.66, %CV = 3.59; ICC = 0.971) and distal phalanx-I (t = -4.488, p<0.01; TEM = 2.49, %CV = 9.68; ICC = 0.934) in the 20-bone protocol, and only for the trapezium (t = -2.921, p<0.01; TEM = 2.46, %CV = 2.43; ICC = 0.987) with the CARPAL protocol. Significant intra-individual differences were absent with the RUS protocol. Agreement rates for bone stages between observers A and B are presented in Table 4. Overall, they were greater than 80% with the 20-bone protocol, but significant differences were noted for 13 bones (5 carpals, metacarpal-I, metacarpal-III, metacarpal-V, proximal phalanges-I, III and V, distal phalanx-III). Bone-specific ICC coefficients ranged from 0.791 to 0.974. The lack of concordance between observers was similar for the CARPAL and RUS systems. Divergence between observers was noted for four of the seven CARPALS and for eight of 13 bones in the RUS system. However, the ICC coefficients for the total scores for each system were 0.990 (20-bone), 0.969 (CARPAL), and 0.988 (RUS).

**Table 1:** Frequencies of developmental stages for each bone assessed with TW2 method assigned by observers A and B on two occasions (time moment 1 *versus* time moment 2), absolute and relative agreement rates, and intra-observer error in assessments of SA among 142 adolescent male soccer players.

Bone	frequencies by stages according to TW2 method																Agreements f (%)	disagreements (stage differences)			
	time moment 1								time moment 2												
	B	C	D	E	F	G	H	I	B	C	D	E	F	G	H	I		-2	-1	+1	+2
**Intra-observer (A)**																					
Radius					4	69	40	29					1	71	43	27	130(91.5%)		5	7	
Ulna			2	4	55	63	18				2	4	50	69	17		132(93.0%)		3	7	
Capitate					1	3	138						1	7	134		138(97.2%)		4		
Hamate					5	10	55	72					5	9	56	72	133(93.7%)		4	5	
Triquetral				14	20	42	66					14	21	41	66		137(96.5%)		3	2	
Lunate				1	15	36	90					1	16	31	94		137(96.5%)		1	4	
Scaphoid			2	7	18	41	74				1	8	19	41	73		133(93.7%)		5	4	
Trapezium			1	2	17	34	46	42			1	3	17	34	45	42	137(96.5%)		4	1	
Trapezoid			1	1	9	53	78				1	1	8	53	79		134(94.4%)		3	5	
Metacarpal I			3	32	21	60	15	11			2	29	27	58	15	11	135(95.1%)		2	5	
Metacarpal III				10	51	52	20	9				7	54	52	20	9	139(97.9%)			3	
Metacarpal V				32	23	62	19	6				32	22	62	19	7	137(96.5%)		1	4	
Proximal phalange I				12	56	59	7	8				11	58	58	7	8	138(97.2%)		2	2	
Proximal phalange III				17	58	40	18	9				15	61	41	17	8	134(94.4%)		5	3	
Proximal phalange V				26	48	47	13	8				26	50	46	12	8	137(96.5%)		4	1	
Medial phalange III				26	43	58	10	5				21	50	56	9	6	134(94.4%)		2	6	
Medial phalange V				46	43	38	10	5				43	52	32	8	7	131(92.3%)		6	5	
Distal phalange I				8	64	42	12	16				8	66	41	11	16	139(97.9%)		3		
Distal phalange III					64	56	13	9					69	52	10	11	134(94.4%)		6	2	
Distal phalange V				8	63	53	8	10				9	65	50	8	10	132(93.0%)		7	3	
All bones n%																	2701		70	69	
																	95.1%		2.5%	2.4%	
**Intra-observer (B)**																					
Radius					5	67	50	20					9	63	53	17	127(89.4%)		11	4	
Ulna				10	48	54	30					11	47	54	30		131(92.3%)		7	4	
Capitate					1	5	136							10	132		135(95.1%)		5	2	
Hamate					3	16	42	81					3	13	45	81	131(92.3%)		4	7	
Triquetral				16	30	38	58					15	27	44	56		133(93.7%)		3	6	
Lunate				2	18	39	83						21	43	78		126(88.7%)		10	6	
Scaphoid			1	16	28	26	71				1	14	26	31	70		131(92.3%)		3	8	
Trapezium			1	4	27	31	41	38			1	4	26	31	41	39	135(95.1%)		2	5	
Trapezoid				2	17	43	80					2	17	43	80		134(94.4%)		4	4	
Metacarpal I			2	7	53	53	16	11			2	6	53	56	14	11	136(95.8%)		3	3	
Metacarpal III				1	46	63	23	9				1	49	60	23	9	137(96.5%)		4	1	
Metacarpal V				17	34	70	15	6				16	34	71	15	6	136(95.8%)		2	4	
Proximal phalange I				7	57	62	7	9				3	67	54	8	10	129(90.8%)		6	7	
Proximal phalange III				7	61	46	18	10				7	57	52	16	10	134(94.4%)		3	5	
Proximal phalange V				20	50	44	17	11				20	53	42	15	12	133(93.7%)		6	3	
Medial phalange III				18	62	44	10	8				18	63	45	8	8	135(95.1%)		5	2	
Medial phalange V				42	64	23	7	9				41	64	23	5	9	133(93.7%)		6	3	
Distal phalange I				24	54	30	15	19				21	57	30	15	19	137(96.5%)		1	4	
Distal phalange III					65	51	11	15					64	53	10	15	136(95.8%)		3	3	
Distal phalange V				5	74	43	6	14				5	76	38	9	14	135(95.1%)		3	4	
All bones n%																	2664		91	85	
																	93.8%		3.2%	3.0%	

TW2 (Tanner-Whitehouse version 2); f (frequencies).

**Table 2:** Descriptive statistics (mean ± standard deviation) for the scores of each bone in the three scoring systems (TW2 20-bone, Carpal, RUS) assigned by observer A on two occasions (time moment 1 *versus* time moment 2), paired t-tests, effect sizes, technical errors of measurement, coefficients of variation and intra-class correlation coefficients among the 142 adolescent male soccer players.

Yi: dependent variable	descriptive statistics		Paired t-test		magnitude effect		TEM	%CV	ICC
	TM1	TM2	t	p	d	‡			
**20-bone**									
Capitate	115±5	114±7	2.022	0.045	0.131	trivial	3.20	2.79	0.823
Hamate	98±10	98±10	-0.312	0.756	-0.008	trivial	2.09	2.14	0.976
Triquetral	50±12	50±12	0.259	0.796	0.004	trivial	1.60	3.18	0.991
Lunate	53±9	54±9	-1.709	0.090	-0.042	trivial	1.96	3.67	0.978
Scaphoid	49±10	48±10	0.422	0.674	0.011	trivial	2.24	4.62	0.976
Trapezium	47±9	46±10	0.935	0.352	0.014	trivial	1.21	2.59	0.992
Trapezoid	48±10	48±10	-0.600	0.549	-0.018	trivial	2.37	4.96	0.968
Radius	87±14	88±12	-1.189	0.237	-0.046	trivial	4.30	4.90	0.943
Ulna	66±12	67±12	-1.423	0.157	-0.045	trivial	3.10	4.66	0.964
Metacarpal-I	26±5	26±4	-1.434	0.154	-0.031	trivial	0.83	3.24	0.984
Metacarpal_III	19±4	19±4	-1.744	0.083	-0.032	trivial	0.62	3.18	0.988
Metacarpal-V	19±4	19±4	-0.930	0.354	-0.014	trivial	0.51	2.72	0.992
Proximal phalange-I	25±4	25±4	-0.203	0.839	-0.005	trivial	0.87	3.46	0.978
Proximal phalange-III	21±3	21±3	-0.610	0.543	-0.015	trivial	0.68	3.25	0.979
Proximal phalange-V	20±4	20±4	0.733	0.465	0.014	trivial	0.57	2.89	0.987
Medial phalange-III	20±4	20±3	-1.845	0.067	-0.054	trivial	0.84	4.28	0.968
Medial phalange-V	18±3	18±3	-0.496	0.621	-0.011	trivial	0.60	3.26	0.981
Distal phalange-I	26±5	26±5	1.514	0.132	0.021	trivial	0.59	2.24	0.993
Distal phalange-III	19±3	19±3	1.918	0.057	0.054	trivial	0.69	3.62	0.971
Distal phalange-V	18±3	18±3	1.417	0.159	0.041	trivial	0.71	3.99	0.969
20-bone score	843±105	844±103	-0.941	0.348	-0.009	trivial	8.01	0.95	0.997
**Carpal**									
Capitate	212±10	211±13	2.022	0.045	0.128	trivial	6.41	3.03	0.834
Hamate	184±14	185±14	-0.333	0.740	-0.011	trivial	3.91	2.12	0.958
Triquetral	105±22	105±22	0.407	0.684	0.006	trivial	2.61	2.48	0.993
Lunate	111±13	111±13	-1.422	0.157	-0.031	trivial	2.47	2.22	0.982
Scaphoid	104±15	104±14	0.252	0.802	0.006	trivial	2.82	2.70	0.981
Trapezium	104±13	103±13	1.527	0.129	0.022	trivial	1.64	1.59	0.992
Trapezoid	103±14	104±14	-0.672	0.503	-0.019	trivial	3.35	3.24	0.972
Carpal score	924±78	923±78	1.117	0.266	0.015	trivial	8.99	0.97	0.993
**RUS**									
Radius	126±50	126±48	0.646	0.520	0.017	trivial	10.55	8.38	0.976
Ulna	94±40	95±39	-0.851	0.396	-0.022	trivial	8.50	9.01	0.977
Metacarpal-I	34±13	35±13	-0.374	0.709	-0.004	trivial	1.11	3.22	0.996
Metacarpal_III	28±11	29±11	-1.744	0.083	-0.014	trivial	0.72	2.53	0.998
Metacarpal-V	27±11	27±11	-1.635	0.104	-0.016	trivial	0.88	3.27	0.997
Proximal phalange-I	34±12	34±12	0.685	0.495	0.007	trivial	1.04	3.07	0.996
Proximal phalange-III	28±10	28±9	0.668	0.505	0.012	trivial	1.42	5.02	0.988
Proximal phalange-V	26±9	26±9	1.178	0.241	0.014	trivial	0.91	3.47	0.995
Medial phalange-III	27±9	28±9	-1.657	0.100	-0.026	trivial	1.22	4.46	0.991
Medial phalange-V	25±9	25±9	1.206	0.230	0.023	trivial	1.53	6.12	0.986
Distal phalange-I	35±13	35±13	1.717	0.088	0.017	trivial	1.11	3.17	0.996
Distal phalange-III	25±8	25±9	0.536	0.593	0.013	trivial	1.77	6.98	0.978
Distal phalange-V	24±9	24±9	1.196	0.234	0.032	trivial	1.94	8.09	0.974
RUS score	535±182	535±180	0.302	0.763	0.003	trivial	13.92	2.60	0.997

TW2 (Tanner-Whitehouse version 2); t (t-value of paired t-test); p (significance value); d (d-cohen value);

^‡^ (qualitative interpretation);

TEM (technical error of measurement); %CV (coefficient of variation); ICC (intra-class correlation coefficient).

**Table 3:** Descriptive statistics (mean ± standard deviation) for each bone score with the three scoring systems (TW2 20-bone, Carpal, RUS) assigned by observer B on two occasions (time moment 1 *versus* time moment 2), paired t-tests, effect sizes, technical errors of measurement, coefficients of variation and intra-class correlation coefficients in 142 adolescent male soccer players.

Yi: dependent variable	descriptive statistics		Paired t-test		magnitude effect		TEM	%CV	ICC
	TM1	TM2	t	p	d	‡			
**20-bone**									
Capitate	115±6	114±7	1.471	0.143	0.108	trivial	3.97	3.47	0.764
Hamate	98±10	99±10	-0.781	0.436	-0.024	trivial	2.50	2.54	0.966
Triquetral	49±12	49±12	-0.105	0.917	-0.002	trivial	2.26	4.66	0.982
Lunate	52±10	52±10	1.330	0.186	0.051	trivial	3.13	6.04	0.946
Scaphoid	47±11	47±11	-0.673	0.502	-0.014	trivial	1.94	4.10	0.985
Trapezium	45±10	45±10	-0.878	0.381	-0.018	trivial	1.69	3.72	0.985
Trapezoid	48±10	48±10	0.000	1.000	0.000	trivial	2.18	4.59	0.976
Radius	87±13	86±15	1.696	0.092	0.073	trivial	5.14	5.96	0.928
Ulna	67±13	67±13	0.364	0.717	0.009	trivial	2.76	4.12	0.977
Metacarpal-I	26±4	26±4	-0.661	0.510	-0.010	trivial	0.45	1.70	0.992
Metacarpal_III	20±3	20±3	1.728	0.086	0.039	trivial	0.62	3.07	0.982
Metacarpal-V	19±3	19±3	-1.000	0.319	-0.019	trivial	0.53	2.76	0.988
Proximal phalange-I	26±4	26±4	-0.243	0.808	-0.010	trivial	1.22	4.73	0.941
Proximal phalange-III	22±3	22±3	-1.590	0.114	-0.026	trivial	0.38	1.74	0.991
Proximal phalange-V	20±3	20±3	0.602	0.548	0.017	trivial	0.79	3.94	0.973
Medial phalange-III	20±3	20±3	0.728	0.468	0.011	trivial	0.41	2.06	0.992
Medial phalange-V	18±3	19±3	-3.754	<0.001	-0.102	trivial	0.66	3.59	0.971
Distal phalange-I	25±6	26±8	-4.488	<0.001	-0.178	trivial	2.49	9.68	0.934
Distal phalange-III	19±3	19±3	-0.355	0.723	-0.009	trivial	0.67	3.48	0.974
Distal phalange-V	18±3	18±3	0.609	0.543	0.013	trivial	0.49	2.71	0.985
20-bone score	841±106	841±105	0.208	0.836	0.002	trivial	9.68	1.15	0.996
**Carpal**									
Capitate	212±12	210±14	1.405	0.162	0.103	trivial	8.01	3.80	0.765
Hamate	185±14	185±14	-1.127	0.262	-0.034	trivial	3.48	1.88	0.967
Triquetral	102±22	103±22	-0.873	0.384	-0.018	trivial	3.73	3.65	0.985
Lunate	109±14	109±13	0.771	0.442	0.031	trivial	4.61	4.23	0.941
Scaphoid	101±17	102±16	-1.460	0.147	-0.031	trivial	2.94	2.89	0.984
Trapezium	101±16	101±14	-2.921	0.004	-0.055	trivial	2.46	2.43	0.987
Trapezoid	103±15	103±15	0.000	1.000	0.000	trivial	3.23	3.14	0.977
Carpal score	912±86	912±83	-0.339	0.735	-0.006	trivial	12.05	1.32	0.990
**RUS**									
Radius	122±45	119±43	1.760	0.080	0.054	trivial	11.44	9.49	0.965
Ulna	101±47	101±47	0.087	0.931	0.003	trivial	12.17	12.18	0.965
Metacarpal-I	35±12	35±12	0.103	0.918	0.002	trivial	1.72	4.90	0.990
Metacarpal_III	30±10	30±10	1.345	0.181	0.025	trivial	1.59	5.29	0.987
Metacarpal-V	27±10	27±10	-0.539	0.591	-0.011	trivial	1.65	6.09	0.986
Proximal phalange-I	35±12	35±12	-0.373	0.710	-0.010	trivial	2.54	7.31	0.975
Proximal phalange-III	29±9	30±9	-0.580	0.563	-0.011	trivial	1.43	4.84	0.987
Proximal phalange-V	27±10	27±10	0.970	0.334	0.017	trivial	1.47	5.37	0.989
Medial phalange-III	27±10	27±9	1.176	0.241	0.024	trivial	1.62	5.93	0.985
Medial phalange-V	25±10	24±9	1.122	0.264	0.023	trivial	1.59	6.46	0.985
Distal phalange-I	34±15	35±15	-1.407	0.162	-0.013	trivial	1.14	3.31	0.997
Distal phalange-III	26±10	26±10	-0.172	0.864	-0.003	trivial	1.37	5.26	0.990
Distal phalange-V	24±9	24±9	-0.138	0.891	-0.002	trivial	1.29	5.32	0.991
RUS score	543±186	540±183	1.194	0.234	0.015	trivial	19.96	3.68	0.994

TW2 (Tanner-Whitehouse version 2); t (t-value of paired t-test); p (significance value); d (d-cohen value);

^‡^ (qualitative interpretation);

TEM (technical error of measurement); %CV (coefficient of variation); ICC (intra-class correlation coefficient).

**Table 4:** Descriptive statistics (mean ± standard deviation) for each bone score in the three scoring systems (TW2 20-bone, Carpal, RUS) assigned by observers A and B, paired t-test, effect sizes, technical errors of measurement, coefficients of variation and intra-class correlation coefficients in 142 adolescent male soccer players.

Variable	% agr.	descriptive statistics		Paired t-test		magnitude effect		TEM	%CV	ICC
		Observer A	Observer B	t	p	d	‡			
**20-bone**										
Capitate	95	114±7	114±7	0.656	0.513	0.046	trivial	3.97	3.47	0.791
Hamate	87	98±10	99±10	-2.347	0.020	0.093	trivial	3.24	3.30	0.940
Triquetral	86	50±12	49±12	4.124	0.000	0.135	trivial	2.26	4.66	0.956
Lunate	83	54±9	52±10	3.968	0.000	0.207	small	4.38	8.34	0.884
Scaphoid	83	48±10	47±11	4.420	0.000	0.111	trivial	2.41	5.04	0.974
Trapezium	82	46±10	45±10	3.982	0.000	0.108	trivial	2.32	5.04	0.971
Trapezoid	81	48±10	48±10	0.647	0.519	0.031	trivial	3.94	8.26	0.912
Radius	80	88±12	86±15	3.219	0.002	0.173	trivial	6.32	7.27	0.880
Ulna	80	67±12	67±13	-0.553	0.581	0.024	trivial	4.49	6.72	0.930
Metacarpal-I	80	26±4	26±4	-4.153	0.000	0.177	trivial	1.52	5.85	0.925
Metacarpal_III	82	19±4	20±3	-4.464	0.000	0.205	small	1.49	7.49	0.910
Metacarpal-V	80	19±4	19±3	-3.962	0.000	0.156	trivial	1.32	6.91	0.937
Proximal phalange-I	87	25±4	26±4	-2.622	0.010	0.122	trivial	1.52	5.98	0.915
Proximal phalange-III	81	21±3	22±3	-4.573	0.000	0.226	small	1.34	6.31	0.894
Proximal phalange-V	80	20±4	20±3	-3.122	0.002	0.122	trivial	1.17	5.95	0.940
Medial phalange-III	80	20±3	20±3	0.346	0.730	0.015	trivial	1.20	6.05	0.925
Medial phalange-V	82	18±3	19±3	-1.890	0.061	0.081	trivial	1.01	5.51	0.930
Distal phalange-I	80	26±5	26±8	-0.327	0.744	0.020	trivial	3.25	12.36	0.854
Distal phalange-III	87	19±3	19±3	-2.183	0.031	0.089	trivial	1.02	5.36	0.936
Distal phalange-V	80	18±3	18±3	-0.262	0.793	0.012	trivial	1.13	6.30	0.917
20-bone score		844±103	841±105	2.027	0.045	0.034	trivial	14.79	1.76	0.990
**Carpal**										
Capitate		211±13	210±14	0.599	0.550	0.042	trivial	8.01	3.81	0.792
Hamate		185±14	185±14	-0.967	0.335	0.054	trivial	6.38	3.45	0.877
Triquetral		105±22	103±22	4.290	0.000	0.122	trivial	5.47	5.27	0.967
Lunate		111±13	109±13	3.577	0.000	0.183	trivial	5.97	5.42	0.891
Scaphoid		104±14	102±16	4.685	0.000	0.150	trivial	4.44	4.30	0.957
Trapezium		103±13	101±14	4.334	0.000	0.129	trivial	3.70	3.62	0.964
Trapezoid		104±14	103±15	1.149	0.253	0.054	trivial	5.84	5.66	0.915
Carpal score		923±78	912±83	4.871	0.000	0.133	trivial	19.78	2.16	0.969
**RUS**										
Radius		126±48	119±43	3.024	0.003	0.134	trivial	17.64	14.41	0.921
Ulna		95±39	101±47	-2.637	0.009	0.136	trivial	19.27	19.72	0.892
Metacarpal-I		35±13	35±12	-2.065	0.041	0.041	trivial	2.09	6.02	0.986
Metacarpal_III		29±11	30±10	-4.000	0.000	0.142	trivial	3.28	11.20	0.949
Metacarpal-V		27±11	27±10	-0.876	0.383	0.027	trivial	2.64	9.79	0.966
Proximal phalange-I		34±12	35±12	-3.080	0.002	0.091	trivial	2.98	8.68	0.966
Proximal phalange-III		28±9	30±9	-4.529	0.000	0.147	trivial	2.63	9.09	0.957
Proximal phalange-V		26±9	27±10	-3.785	0.000	0.120	trivial	2.71	10.14	0.960
Medial phalange-III		28±9	27±9	1.186	0.238	0.046	trivial	3.01	10.99	0.944
Medial phalange-V		25±9	24±9	1.182	0.239	0.042	trivial	2.77	11.22	0.954
Distal phalange-I		35±13	35±15	0.820	0.414	0.024	trivial	3.54	10.17	0.968
Distal phalange-III		25±9	26±10	-2.808	0.006	0.095	trivial	2.68	10.44	0.956
Distal phalange-V		24±9	24±9	-1.082	0.281	0.043	trivial	3.02	12.57	0.941
RUS score		535±180	540±183	-1.751	0.082	0.031	trivial	27.45	5.11	0.988

TW2 (Tanner-Whitehouse version 2); t (t-value of paired t-test); p (significance value); d (d-cohen value);

^‡^ (qualitative interpretation);

TEM (technical error of measurement); %CV (coefficient of variation); ICC (intra-class correlation coefficient).

**Fig 1:**
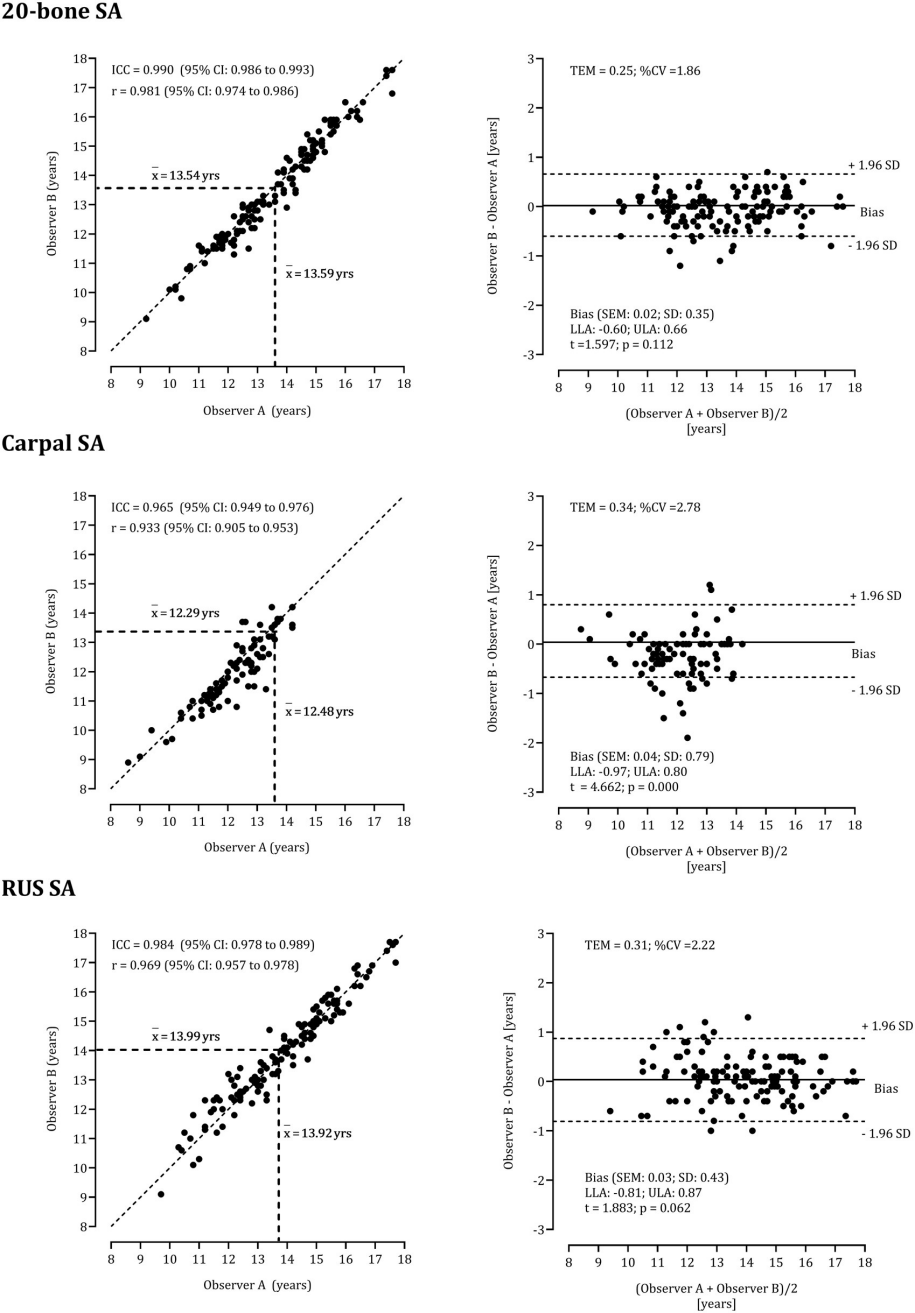
Interobserver agreement for the determination of skeletal age by two observers, considering concurrent systems (20-bone, Carpal and RUS) and indication of the mean for each observer, bivariate correlation coefficient between the series produced by observers A and B, intra-class correlation coefficient; complemented with Bland-Altman analysis to inspect intra-individual differences expressed against the mean values.

The impact of observer-associated variation in the point scores for each protocol on the respective SAs is illustrated in Fig 1. Comparisons between observers were not significant for 20-bone SAs (TEM = 0.25 years, %CV = 1.86; ICC = 0.990, 95%CI: 0.986 to 0.993) neither the RUS SAs (TEM = 0.31 years, %CV = 2.22; ICC = 0.984, 95%CI: 0.978 to 0.989). Although the difference between mean CARPAL SAs of the two observers was small, 12.48±1.18 years and 12.29±1.24 years for observers A and B, respectively (t = 4.662, p<0.01), the difference between observers had little impact as shown in the respective panel: ICC = 0.965, 95%CI: 0.949 to 0.976, TEM = 0.34 years, %CV = 2.78). For the three SA protocols, BIAS was negligible: 0.02 years (20-bone), 0.04 years (CARPAL), and 0.03 years (RUS).

## Discussion

The present study evaluated intra-observer agreement for SA assessments on two independent occasions using the TW2 20-bone, CARPAL and RUS protocols among male soccer players 11–15 years of age. Overall agreement between the two time-moments was acceptable for the three systems. Discrepancies did not exceed one stage and there was no specific trend for the replicate assessment to exceed or fall below that for the initial assessment. With the 20-bone protocol, bone-specific technical errors of measurement were always < 5% of one observer and exceeded 5% for only three bones by the other observer. Disagreement seemed slightly higher for the CARPAL and RUS protocols which are based on smaller number of bones; this likely reflected the scoring system as the 20 bone, CARPAL and RUS protocols were based on a 1000-point scale. Nevertheless, allowing for several problematic bones, intra-observer agreement for the respective SAs were acceptable both in terms of scores and assigned SAs.

TW2 protocol has been updated (TW3) and has been used in the sports sciences [11,21,22]. The original version (TW1) was developed on a British sample of average socioeconomic status [6]. The scores were designed to represent biological weights for each of 20 bones bone and the overall score was obtained by summing the scores of the 20 bones. Specific tables were used to convert the 20 bone score into a SA (20-bone TW1-SA). The first revision of the method (TW2) retained the verbal criteria for the respective stages of the 20 bones with few refinements [7]: radius (stage J was deleted), ulna (stages I was deleted) and for five carpals (capitate, triquetral, lunate, scaphoid, trapezoid) the final stage I was deleted. This initial revision included changes in the scores associated with each stage. Three maturity scores were separately developed for boys and girls to derive an SA with each protocol: carpals (CARPAL TW2 -SA), radius, ulna and short bones (RUS TW2 -SA) in addition to the 20-bone TW2SA.

The most recent revision for the TW protocol (TW3) incorporated several additional samples of children and adolescents in revising the tables for converting the CARPAL and RUSs into SAs [8]. The British samples of the initial study dated from 1950s was retained while samples from Belgium (Leuven Growth Study in the 1970s) [23], Spain (Bilbao in the 1980s) [24], Japan (Tokyo in 1986) [25], Italy (north of Italy) [26], Argentina (LaPlata in the 1970s) [27], and the U.S. (Texas, European-American ancestry) [28] were added.

The specific stages and corresponding scores were the same as in TW2, but the TW3 revision deleted the 20-bone SA. As such, the TW3 revision includes only sex-specific tables CARPAL TW3-SA and RUS TW3-SA. In addition, skeletal maturity for the RUS TW3 protocol is attained at 16.5 years for males and 15.0 years for females. In the preceding versions of the TW method, the pre-mature state (999 points) for males corresponded to an SA of 17.9 years with the TW2 20-bone, 18.1 years with the TW2-RUS and 14.9 years with the TW2-CARPAL scoring protocols.

Early studies reporting intra-observer agreement of the TW2 method date to 1970s. In a sample of Swedish 122 boys and 90 girls 1 month to 7 years of age, replicate assessments had an overall agreement rate of about 80% [29]. Among 3817 Danish school children 7 to 18 years TW 20 bone scores largely matched the British reference [30]. In the preceding study, 90 radiographs were rated twice and agreement rates were 88–89% for the long bones and 84–96% for the short bones. Since the carpals attained the final stages at earlier ages compared to long bones, the Danish study decided to examine x-rays from 7–13 years old boys and 7–11 years old girls, in a total of 60 cases, to obtain an agreement rate ranging 82–93%.

Meantime, TW2 assessments was previously carried out using three observers [31]. Two sets of x-rays in a random order obtained from the Harpenden Longitudinal Growth Study and from the Leuven Longitudinal Study of Belgian Boys were used to test the agreement rates between observers. Significant differences were found in mean SA between observers for 20-bone SA and CARPAL SA. In contrast, no significant differences in mean SA between observers were found for RUS SA. In the present study, after converting scores to SAs, inter-observer mean differences were not significant for the TW2-20bone and for TW2-RUS SAs. In contrast, the inter-observer difference with the TW2-CARPAL protocol was a source of error with 15 cases exceeding the limits of agreement in the present study. Among 110 Danish children and adolescents aged 6–16 years [32], intra-observer agreement fluctuated between 82% to 100%, and consistent with the current study, disagreements did not exceed more than one stage with capitate diagnosed as the most critical bone for disagreement.

Inter-observer agreement rates TW SA assessment are less frequently reported in the literature compared to intra-observer differences. In a study of Dutch children [33], 60 radiographs of boys 10 through 16 years were rated with the TW protocol by an expert and a Dutch author. The percentage of agreement for the ulna was 83% and that for the radius 66% with a systematic disagreement that was concentrated in the assessment of stage F. This prompted the authors to hypothesize a differential impact of observer expertise among youth 10–12 years of age. Meantime, in the present study of soccer players, disagreements between observers that exceeded the limits of agreement were concentrated between 11–13 years for TW2 Carpal SA and between 11.5–14.0 years for TW2 20-bone SA (see Fig 1).

The literature on the skeletal maturity status of youth soccer players has consistently shown that the sport tends to favor early maturing players as they transition into the adolescent years [3,9]. A band of plus/minus 1.0 year is commonly used to classify players as late, average or early maturing. In the present study and based on assessments of observer A (first author), early maturing players represented 36% at time moment 1 (TM1) and 37% (TM2) while using the TW-2 20-bone SA, thus suggesting that intra-individual error marginally impacted the frequencies of maturity status. Corresponding estimates of maturity classifications with TW2-RUS SA classified 49% and 50% of the participants as advanced in TM1 and TM2, respectively. In contrast, percentages of players classified as advanced with TW2-CARPAL SA were, respectively, 8% and 11%. By inference, intra-observer error in assessments did not appear to influence maturity status classifications.

The present study highlights the expertise of SA assessments with the TW2 protocol among adolescent soccer players. The study is novel as it considers intra-observer analyses for each bone in addition to the three protocols (20-bone, CARPALS, RUS) both using scores and assigned SAs as the dependent variable. Nevertheless, few limitations should be considered. First, the study was focused on the ability of two observers and inter-examiner agreement is essential in research projects using more than two examiners. Additionally, the results are limited to a sample of 142 male soccer players 11–15 years. Given the CA range, it was not possible to evaluate early stages for specific boys, e.g., stages B-E for the radius, capitate, hamate and distal phalange III; B-D for the triquetral, lunate, metacarpals II-V, proximal phalanges I-V, and distal phalanges I and V; and for stages B-C of the ulna, scaphoid, trapezium, trapezoid, and metacarpal I. By inference, there is a need for additional research on pre-teens, especially for CARPAL protocol. Note, the age interval of the current sample included 25 participants who were classified as skeletally mature and as such were not included in the calculations illustrated in Fig 1. Nevertheless, the literature generally considers descriptors of the stages for round bones (carpals) more difficult to evaluate compared to long bones and as noted, the capitate has been previously indicated as problematic [31,34,35]. The carpals are more difficult to evaluate because they involve assessments of shape and radiopaque lines or zones, whereas assessments of the long bones tend to concentrate on the centers of ossification and epiphyseo-diaphysial relationships and fusion [36].

## Conclusions

In summary, the assignment of developmental stages is specific for each bone and is somewhat more problematic for the round (carpals) than for the long bones. Examiners should be encouraged to evaluate their expertise on perhaps 100 images spanning a broad range of CAs. Data quality using adolescent samples should not be generalized to early ages. Finally, if disagreements between replicate assessments are not greater than one stage and shows equal probability for the replicates to be lower or higher compared to initial assignments, the effect on assigned SAs appears to be trivial or small.

## Supporting information

S1 File (XLSX)
